# Preparation, Characterization, and Immuno-Enhancing Activity of Polysaccharides from *Glycyrrhiza uralensis*

**DOI:** 10.3390/biom10010159

**Published:** 2020-01-19

**Authors:** Adila Aipire, Pengfei Yuan, Alimu Aimaier, Shanshan Cai, Mahepali Mahabati, Jun Lu, Tianlei Ying, Baohong Zhang, Jinyao Li

**Affiliations:** 1 Xinjiang Key Laboratory of Biological Resources and Genetic Engineering, College of Life Science and Technology, Xinjiang University, Urumqi 830046, China; kaskas999@163.com (A.A.); 18690282867m0@sina.cn (P.Y.); alimu15276554161@163.com (A.A.); 18699927521@163.com (S.C.); mahpal13565987204@163.com (M.M.); 2 School of Science, and School of Interprofessional Health Studies, Faculty of Health & Environmental Sciences, Auckland University of Technology, Auckland 1142, New Zealand; jun.lu@aut.ac.nz; 3 Key Laboratory of Medical Molecular Virology of MOE/MOH, Shanghai Medical College, Fudan University, Shanghai 200032, China; tlying@fudan.edu.cn; 4 Engineering Research Center of Cell and Therapeutic Antibody, Ministry of Education; School of Pharmacy, Shanghai Jiao Tong University, Shanghai 200240, China; bhzhang@sjtu.edu.cn

**Keywords:** *Glycyrrhiza uralensis* polysaccharides, molecular weight, monosaccharide composition, structural characterization, immuno-enhancing activity

## Abstract

*Glycyrrhiza uralensis* is a Chinese herbal medicine with various bioactivities. Three fractions (GUPS-I, GUPS-II and GUPS-III) of *G. uralensis* polysaccharides (GUPS) were obtained with molecular weights of 1.06, 29.1, and 14.9 kDa, respectively. The monosaccharide compositions of GUPS-II and GUPS-III were similar, while that of GUPS-I was distinctively different. The results of scanning electron microscopy, FT-IR, and NMR suggested that GUPS-II and GUPS-III were flaky with a smooth surface and contained α- and β-glycosidic linkages, while GUPS-I was granulated and contained only α-glycosidic linkages. Moreover, GUPS-II and GUPS-III exhibited better bioactivities on the maturation and cytokine production of dendritic cells (DCs) in vitro than that of GUPS-I. An in vivo experiment showed that only GUPS-II significantly enhanced the maturation of DCs. These results indicate that GUPS-II has the potential to be used in combination with cancer immunotherapy to enhance the therapeutic effect.

## 1. Introduction

Polysaccharides of natural sources show various bioactivities, such as antitumor [1,2,3], antioxidant [4,5], immunomodulatory [6,7], and anti-inflammatory effects [8], which are closely correlated with their structures and conformations. Many plant-derived polysaccharides have been extensively studied for immune enhancing effect because of their safety profiles [9]. Polysaccharides can activate macrophages, dendritic cells (DCs), T lymphocytes, B lymphocytes, and natural killer cells to produce immune-related molecules, such as cytokines, antibodies, and complement molecules [10,11,12]. Recently, several studies have shown that polysaccharides bind to receptors such as Toll-like receptors (TLRs) on the surface of macrophages or DCs to trigger several down-stream signaling pathways to promote immune responses [13,14]. It has been reported that the chemical structures and chain conformations of polysaccharides are closely correlated to their biological activities [15].

*Glycyrrhiza uralensis*, a member of the Leguminosae family, has been used to treat various diseases such as phlegm, cough, dyspnea, spasms, and pain in traditional herbal medicine [16,17]. A lot of components including triterpene saponins, flavonoids, chalcones, and polysaccharides have been identified from licorice, which exhibit anti-inflammatory, antiviral, antioxidant, antitumor, and immunomodulatory activities [16,18,19,20,21,22]. Polysaccharides are one of the most abundant components in *G. uralensis*, which show immune-enhancing activity. Our previous study showed that *G. uralensis* water extract (contained 25% polysaccharides) enhanced the maturation and function of DCs, and exhibited antitumor efficacy on human papillomavirus (HPV)-DC vaccine [23]. Cheng et al. purified glycyrrhiza polysaccharides and found that the polysaccharides could activate macrophages to increase the pinocytic activity and the production of nitric oxide, interleukin-1 (IL-1), IL-6, and IL-12 [16]. Wu et al. reported that glycyrrhiza polysaccharide liposomes enhanced the function and cytokine production (IL-2 and interferon-γ) of chicken bone marrow-derived DCs compared with glycyrrhiza polysaccharides [18]. However, the structure-activity correlation of *G. uralensis* polysaccharides (GUPS) is still elusive.

In this study, we purified three polysaccharide fractions using a DEAE-52 column and named them GUPS-I, GUPS-II, and GUPS-III. The chemical composition, preliminary structural features, and immune-enhancing activities of these polysaccharides were comparatively investigated. This study provides some hints for the correlation between the structure and activity of GUPS or other polysaccharides from natural sources.

## 2. Materials and Methods

### 2.1. Chemicals and Reagents

DEAE-52 cellulose was obtained from GE Healthcare (Uppsala, Sweden). The monosaccharides, comprising ribose (Rib), glucuronic acid (GlcA), glucose (Glc), galactose (Gla), mannose (Man), arabinose (Ara), rhamnose (Rha) and xylose (Xyl), lipopolysaccharide (LPS), active carbon, trifluoroacetic acids (TFA), and FITC-Dextran, were purchased from Sigma-Aldrich (St Louis, MO, USA). T-series dextrans (T-10, T-40, T-70, T-500 and Blue dextran) were purchased from Solarbio (Beijing, China). Fetal bovine serum (FBS) and Penicillin-streptomycin were purchased from MRC (Changzhou, China). Medium RPMI-1640 and phosphate-buffered solution (PBS) were purchased from Gibco (Grand Island, NY, USA). Granulocyte-macrophage colony-stimulating factor (GM-CSF) was purchased from PeproTech (Rocky Hill, NJ, USA). The other chemical reagents were purchased from Tianjin Fuchen (Tianjin, China). 

The antibodies for flow cytometry comprising anti-CD40-APC, anti-CD86-APC, anti-CD11c-FITC, and ELISA kits including tumor necrosis factor-α (TNF-α), IL-1β, and IL-12p40 were purchased from Elabscience (Wuhan, China). Anti-MHC-I-FITC, anti-MHC-II-PE, and anti-CD11c-PE were bought from BD Biosciences (San Diego, CA, USA).

### 2.2. Preparation of G. uralensis Crude Polysaccharides (GUPS-C)

*G. uralensis* roots were collected from Yili in Xinjiang Uygur autonomous region, China. Polysaccharides were isolated using a previously-described procedure with some modifications [23]. In brief, 100 g of *G. uralensis* minced roots were extracted by 500 mL of petroleum ether twice at 60 °C for 1 h; then, the residues were collected and extracted with 500 mL of 80% ethanol twice at 60 °C for 1 h. The residues was collected and dissolved in 800 mL of distilled water. After treatment with ultrasonication for 20 min, the solution was placed in water bath at 60 °C for 2 h. The step was repeated once. The supernatant was collected and concentrated using a rotary vacuum evaporator at 40 °C, then decolored with 5% active carbon for 30 min. The concentrated solution was precipitated twice with 4 volumes of ethanol at 4 °C for 24 h. After spinning down at 9610 g for 15 min, the GUPS-C was obtained.

### 2.3. Purification of GUPS

GUPS-C was further purified by DEAE-52 chromatography according to our previous protocol with a minor modification [11]. In brief, 0.5 g of GUPS-C was dissolved in 200 mL distilled water and filtered through 0.22 μM filter. The solution was concentrated to 50 mL, and applied to a column (2.6 cm × 20 cm) of DEAE-52 cellulose equilibrated with water. One column volume (CV) is about 106.186 (cm^3^). After loading with the sample, the column was step-wise eluted with 3 CV of distilled water and gradients of NaCl solutions (0.1, 0.2, 0.5, and 1 M) at a flow rate of 1 mL/min. The fractions were collected at 7 min intervals with a fraction collector (ÄKTA Purifier 100, GE, Marlborough, MA, USA) and measured by the phenol-sulfuric acid method. Three major peaks were obtained according to the elution with water, 0.1 M, and 0.2 M NaCl solutions, respectively. The major fractions of each peak were pooled, dialyzed, and freeze-dried to yield the polysaccharide powder, named GUPS-I, GUPS-II, and GUPS-III, respectively. Their yields were 30%, 8%, and 16%, respectively.

### 2.4. Analysis of Carbohydrate, Protein, and Sulfate Contents

Total carbohydrate was determined by the phenol-sulfuric acid method using glucose as the standard [24]. Protein was determined by using BCA Protein Assay Kit (Thermo, Waltham, MA, USA) according to the manufacturer′s instruction. The sulfate contents were determined by BaCl_2_-gelatin assay using potassium sulfate as the standard [25]. Polysaccharide solutions of 1 mg/mL concentration were scanned from 200 nm to 400 nm with a UV spectrophotometer (UV-3600).

### 2.5. Monosaccharide Composition Analysis

The monosaccharide composition of GUPS was analyzed by GC-MS [26]. GlcA, Glc, Gla, Man, Ara, Rha, and Xyl were used as the monosaccharide standards. Briefly, 10 mg of GUPS was hydrolyzed with 4 mL of 2 M TFA at 110 °C for 6 h under a N_2_ atmosphere. The monosaccharides and hydrolyzed products were reacted with 10 mg of NaBH_4_, pyridine (0.5 mL), and acetic anhydride (0.5 mL) at 90 °C for 30 min to prepare the acetylated samples, which were detected by GC–MS with an OV-1701 capillary column (30 m × 0.32 mm). The temperature program was set at 160 °C and then gradually increased to 230 °C at an increment of 5 °C/min, with the carrier gas of high purity He_2_ and flame ionization detector being set at 270 °C. The injection volume was 1 μL.

### 2.6. Molecular Weight Determination

The homogeneity and molecular weight of GUPS were determined by HPGPC according to our previous study [11]. A set of dextran standards with molecular weights of 10, 40, 70, 500, and 2000 kDa were used to make the standard curve. The molecular weight of GUPS was estimated according to the standard curve.

### 2.7. SEM Analysis

For the morphologies of GUPS, the samples were coated with a thin gold layer and observed by scanning electronic microscope (SU8010, Hitachi, Tokyo, Japan). 

### 2.8. Fourier Transform Infrared (FT-IR) Spectroscopy and Nuclear Magnetic Resonance (NMR) Spectroscopy

First, 5 mg of GUPS was mixed with 400 mg of KBr to make a tablet and recorded on an FT-IR spectrometer (Bruker, VERTEX 70, Fallanden, Switzerland) from 4000 to 400 cm^−1^ with 64 scans at 4 cm^−1^ resolution using the OPUS 6.5 software (Bruker).

Approximately 40 mg of GUPS was dissolved in 0.5 mL of D_2_O and then analyzed using a Bruker 600 MHz NMR apparatus (Bruker, Fallanden, Switzerland) at 60 °C. The one-dimensional spectra (^1^H and ^13^C) and two-dimensional spectra (HSQC and HMBC) were measured. Chemical shifts of GUPS were expressed in ppm.

### 2.9. DPPH Radical Scavenging Assay

The DPPH radical scavenging assay was performed according to previous description [27]. Briefly, 50 μL of GUPS fractions at different concentrations (0.05, 1, 2, 3, 4, and 5 mg/mL) were mixed with 150 μL of 0.2 mM DPPH ethanol solution. The ascorbic acid (Vc) was used as positive control. The OD was measured at 517 nm using a microplate reader (Bio-Rad, Hercules, CA, USA). The DPPH radical scavenging capability was calculated according to the equation:

Scavenging rate (%) = [(Ab − As)/Ab] × 100%(1)

Ab is the OD in the absence of the test sample and As is the OD in the presence of the test sample.

### 2.10. Generation and Treatment of Bone Marrow-Derived DCs

Dendritic cells were generated from bone marrow cells of C57BL/6 mice in the presence of GM-CSF following a previously-described protocol [28]. On day 7, DCs were harvested and treated with different concentrations (10, 50 μg/mL) of GUPS for 12 h. LPS (20 ng/mL) were used as the positive control.

For the analysis of phagocytosis of DCs, cells were incubated with FITC-Dextran (Sigma-Aldrich) for 1 h after treatment with GUPS (50 μg/mL) or LPS for 12 h and analyzed by a flow cytometer.

### 2.11. Animal Experiment

C57BL/6 mice (6–8 weeks of age) were bought from Animal Laboratory Center, Xinjiang Medical University (Urumqi, Xinjiang, China) and housed in a temperature-controlled, light-cycled animal facility in Xinjiang University. Mice (3 mice/group) were injected with GUPS-I, GUPS-II, or GUPS-III (100 μg/mice) or LPS (100 ng/mouse) by both sides of footpads (25 μL/footpad). Mice injected with PBS or LPS were used as negative or positive controls. After 24 h, popliteal lymph nodes (LNs) were isolated to analyze DC maturation by using a flow cytometer.

### 2.12. Ethics Statements

All animal experiments were approved by the Committee on the Ethics of Animal Experiments of Xinjiang Key Laboratory of Biological Resources and Genetic Engineering (BRGE-AE001) and carried out under the guidelines of the Animal Care and Use Committee of College of Life Science and Technology, Xinjiang University.

### 2.13. Flow Cytometry

For in vitro analysis of DC maturation, GUPS treated cells were stained with fluorescence-conjugated anti-CD11c, anti-CD40, anti-CD86, and anti-MHC-I/II at room temperature (RT) for 15 min. For the detection of the phagocytosis activity of DCs, cells were stained with anti-CD11c-PE at RT for 15 min and collected by FACSCalibur. For in vivo analysis of DC maturation, the lymphocytes of popliteal LNs were stained with fluorescence-conjugated anti-CD11c, anti-CD40, anti-CD86, and anti-MHC-I/II at RT for 15 min. All samples were collected by FACSCalibur (BD Biosciences) and the data were analyzed using FlowJo software (Tree Star, Inc., Ashland, OR, USA).

### 2.14. ELISA Assay of Cytokines

The supernatants of DC culture were collected and the levels of TNF-α, IL-1β, and IL-12p40 were determined by ELISA kits according to the manufacturer’s instruction. 

### 2.15. Data Analysis

All experiments were carried out at least thrice, and the data were expressed as mean ± SD. Statistical analysis was carried out by one-way analysis of variance (ANOVA) using Prism5.0 software. *p* < 0.05 was considered to be statistically significant.

## 3. Results

### 3.1. Purification and Physicochemical Characterization of GUPS

**Figure 1:**
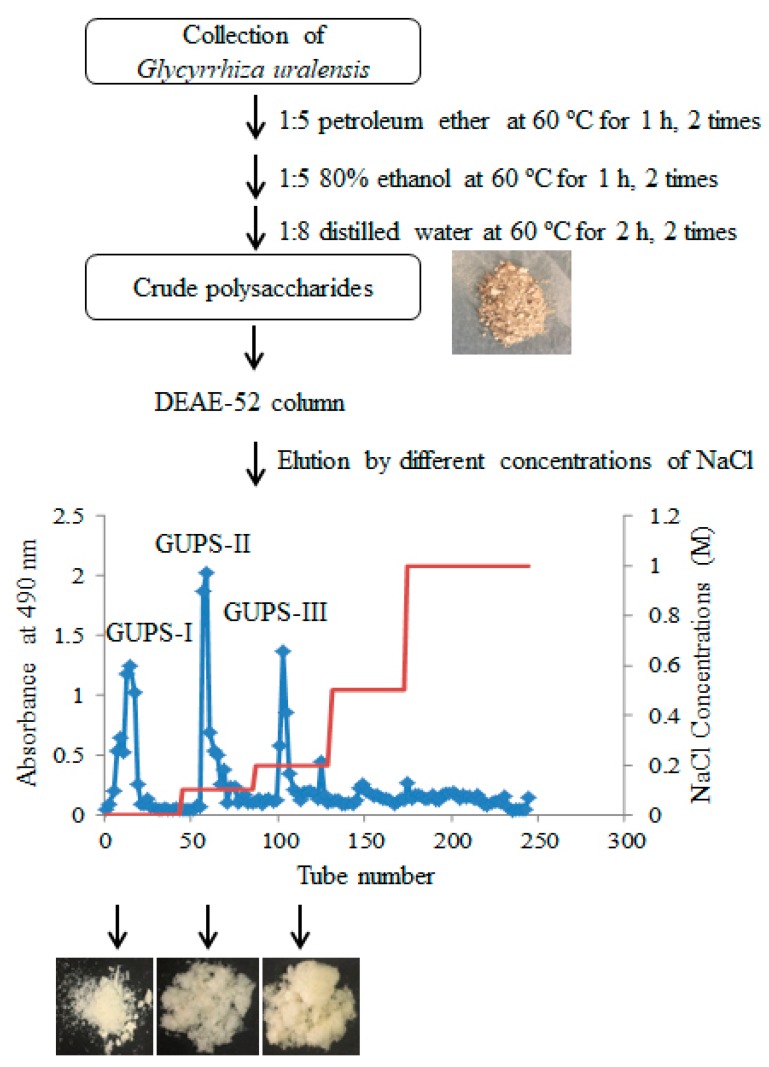
The strategy of GUPS purification.

The crude polysaccharides of *G. uralensis* (GUPS-C) were isolated using the method of hot water extraction and alcohol deposition according to the procedure in Figure 1. The yield of GUPS-C was approximately 2.73% (*w*/*w*). GUPS-C was further separated through cellulose DEAE-52 anion-exchange column with elution by different concentrations of NaCl. As shown in Figure 1, three fractions, i.e., GUPS-I, GUPS-II, and GUPS-III, were collected, concentrated, dialyzed, and lyophilized. GUPS-I eluted with distilled water might be a neutral polysaccharide, whereas both GUPS-II and GUPS-III eluted with NaCl solution might be acidic polysaccharides [29]. 

The physicochemical properties of the GUPS were determined and shown in Table 1. GUPS-I is white and granulated, while GUPS-II and -III are yellow and loose. The molecular weights of GUPS-I, -II, and -III are 1060, 29,100, and 14,900 Da, respectively. The total carbohydrate contents of GUPS-C, GUPS-I, GUPS-II, and GUPS-III were 37%, 29%, 93%, and 75%, respectively. Based on the BCA test, GUPS-I and GUPS-II did not contain protein, but GUPS-C and GUPS-III contained 4% and 21.5% protein, respectively. This is consistent with the result from the UV-Visible spectroscopy of the three fractions (Supplementary Figure S1). The sulfate contents of GUPS-I, GUPS-II, and GUPS-III were 6.5%, 8.7%, and 20.47% respectively.

### 3.2. Monosaccharide Composition of GUPS

**Figure 2:**
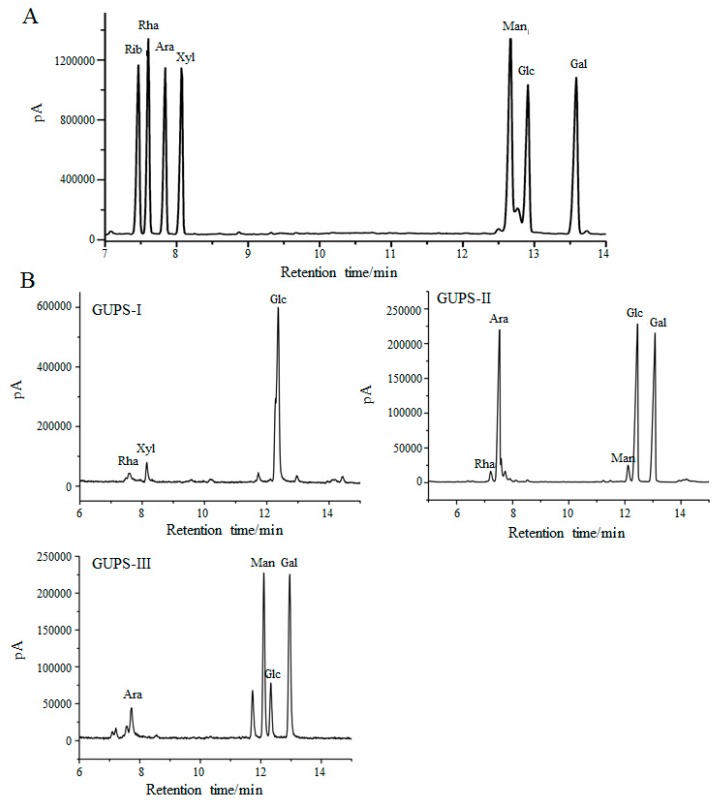
The monosaccharide composition of GUPS. (**A**) The GC-MS of standard monosaccharides. (**B**) The GC-MS of GUPS-I, GUPS-II, and GUPS-III.

The monosaccharide composition of GUPS-I, GUPS-II, and GUPS-III was analyzed by GC-MS and compared with monosaccharide standards. The results showed that GUPS-I was composed of Rha, Xyl, and Glc at molar ratios of 2.55:1.9:30.03, GUPS-II consisted of Rha, Ara, Man, Glc, and Gal at molar ratios of 1:13.87:1.59:16.76:15.72, and GUPS-III was composed of Ara, Man, Glc, and Gal at molar ratios of 1:5.33:1.91:5.97 (Figure 2). The monosaccharide composition of GUPS-II was similar to that determined in previous studies, but GUPS-III had some differences, such as the mannose content [30,31]. 

### 3.3. Scanning Electron Microscopy (SEM) Analysis of GUPS 

**Figure 3:**
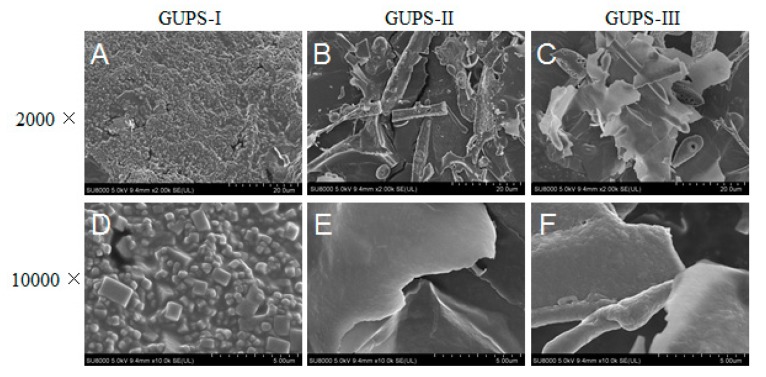
SEM images of GUPS-I, GUPS-II and GUPS-III. The magnifications of (**A–C**) and (**D–F**) are 2000× and 10,000×.

The preliminary structures of GUPS-I, GUPS-II, and GUPS-III were observed using SEM at 2000- and 10,000-fold magnifications (Figure 3). GUPS-I was granulated, suggesting that it is mainly composed of the oligosaccharide. This is consistent with its low molecule weight. However, GUPS-II and GUPS-III were flaky without a porous structure. Furthermore, GUPS-II and GUPS-III showed a smooth surface at 10,000-fold magnification, suggesting that they may form an amorphous structure. 

### 3.4. The Analysis of GUPS by FT-IR Spectroscopy

**Figure 4:**
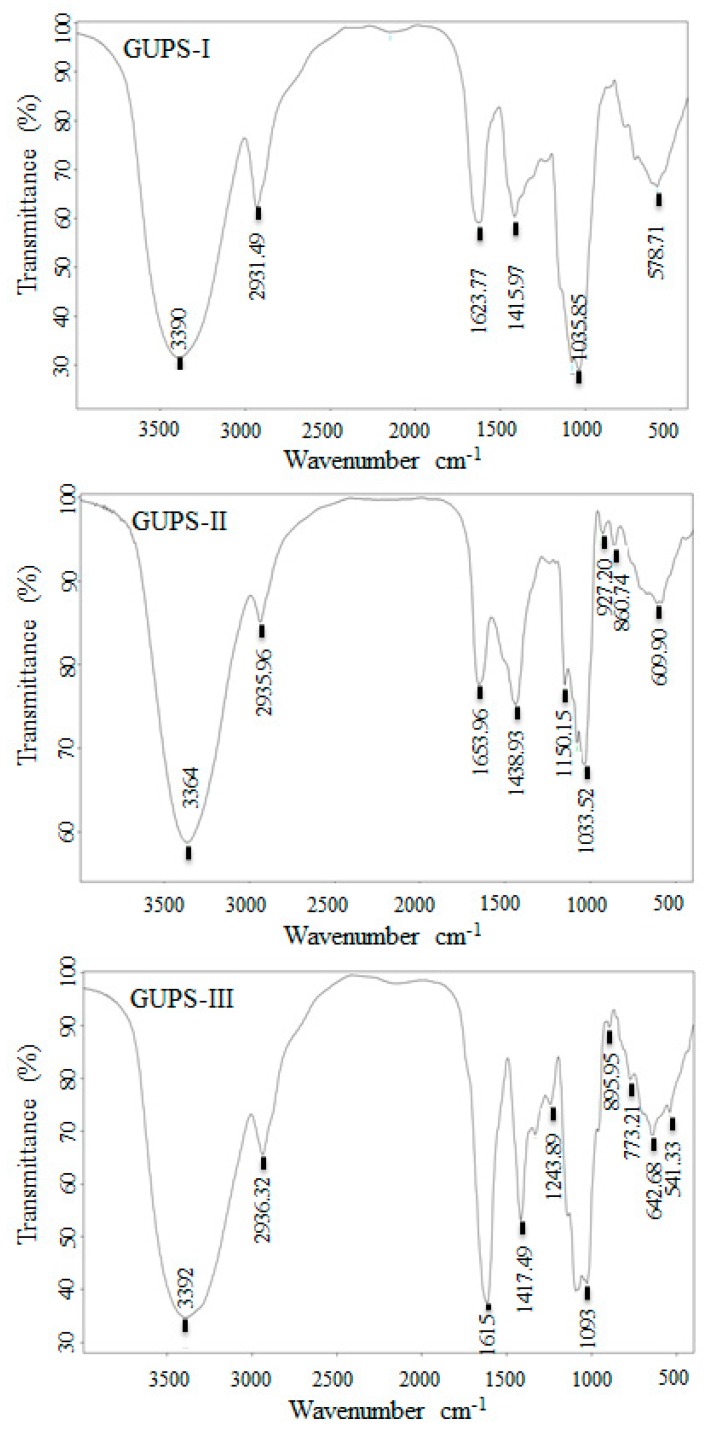
FT-IR spectra of GUPS-I, GUPS-II, and GUPS-III.

FT-IR spectra of carbohydrates are commonly used for the determination of structural features [32]. As shown in Figure 4, the IR spectra of GUPS-I, GUPS-II, and GUPS-III had a broad, intense hydroxyl group stretching band at 3400–3300 cm^−1^ and a weak C-H stretching band at around 3000–2800 cm^−1^, which are the characteristics of polysaccharides [12,33]. In the three spectra, the absorption peaks at approximately 1610–1655 cm^−1^ corresponded to carbonyl groups (C=O) and were caused by the bending mode of bound water, while the absorption peak at approximately 1400 cm^−1^ was the C–O stretching vibration [26,29,34,35]. A strong absorption peak at 1615 cm^−1^ presented carboxyl groups (COO−) in GUPS-III, indicating the existence of uronic acids [36,37]. A strong band between 950 and 1160 cm^−1^ in the three spectra of GUPS were attributed to the stretching vibrations of the pyranose ring [38]. In addition, the peak appeared at approximately 1077 cm^−1^ in the three spectra was the result of the glycosidic linkage stretch vibration of C–O–C bond [34]. The characteristic absorption peaks at 890–920 cm^−1^ in GUPS-II and GUPS-III were due to β-pyranose configuration [5]. Moreover, a weak band at approximately 860 cm^−1^ in GUPS-II was attributed to α-glycosidic linkages in the polysaccharide chains. Another weak band at 1250–1244 cm^−1^ in GUPS-III was regarded as the S=O vibration, suggesting that GUPS-III may consist of sulfated polysaccharides [39,40,41]. This is in consistent with the sulfate content of GUPS-III. 

### 3.5. The NMR Spectrum of GUPS

**Figure 5:**
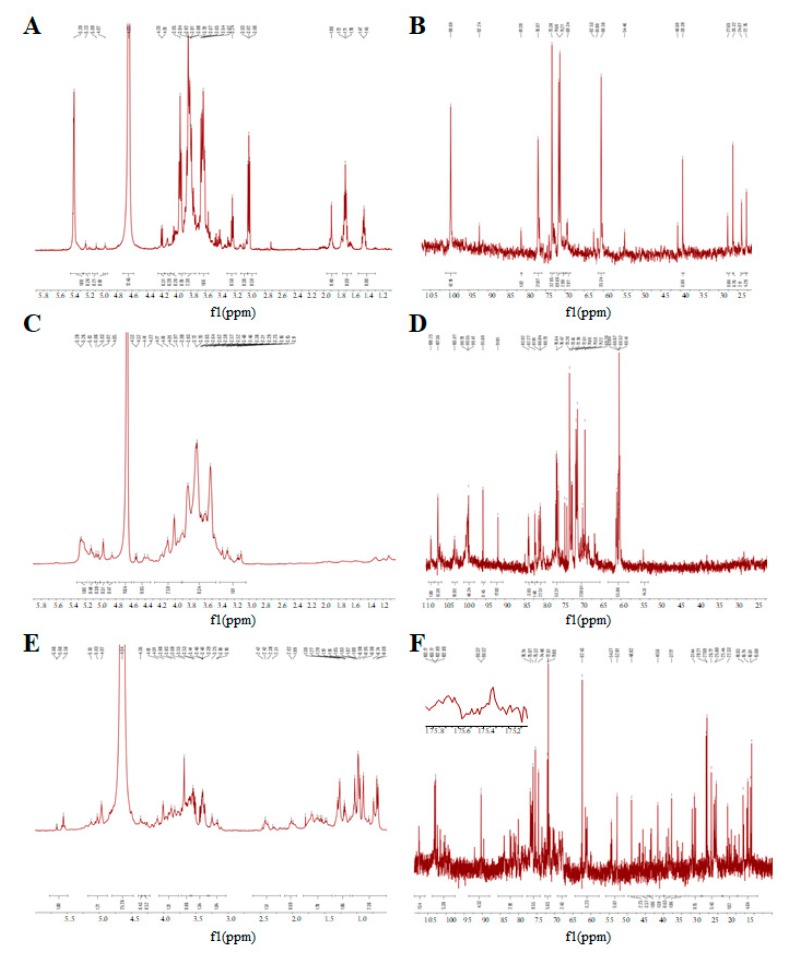
One-dimensional NMR spectra of GUPS. (**A**) ^1^H NMR spectrum of GUPS-I; (**B**) ^13^C NMR spectrum of GUPS-I; (**C**) ^1^H NMR spectrum of GUPS-II; (**D**) ^13^C NMR spectrum of GUPS-II; (**E**) ^1^H NMR spectrum of GUPS-III; (**F**) ^13^C NMR spectrum of GUPS-III.

**Figure 6:**
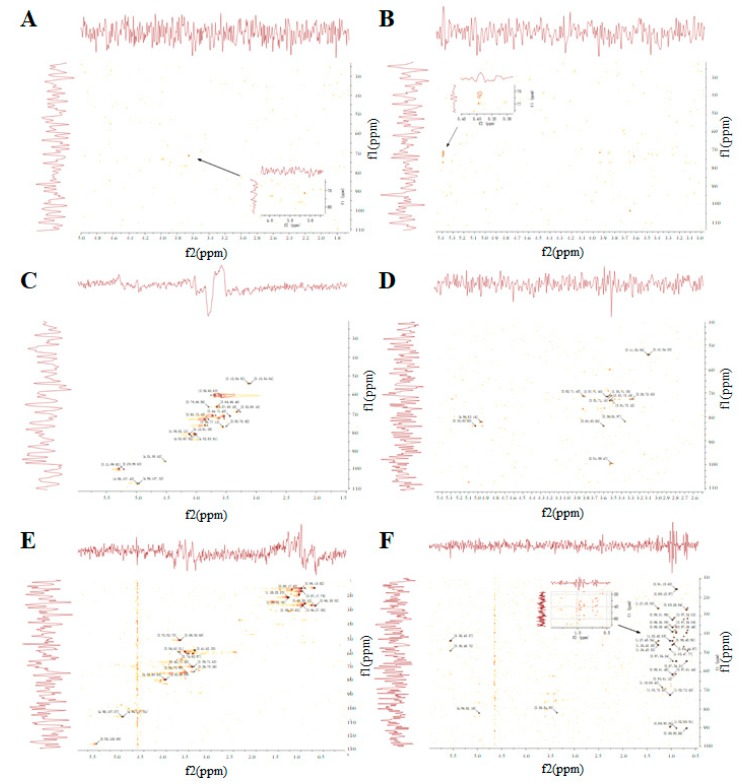
Two-dimensional NMR spectra of GUPS. (**A**) HSQC spectrum of GUPS-I; (**B**) HMBC spectrum of GUPS-I; (**C**) HSQC spectrum of GUPS-II; (**D**) HMBC spectrum of GUPS-II; (**E**) HSQC spectrum of GUPS-III; (**F**) HMBC spectrum of GUPS-III.

NMR spectroscopy is an efficient way to elucidate the chemical constituents of polysaccharides [42]. GUPS-I, GUPS-II and GUPS-III were analyzed via ^1^H NMR, ^13^C NMR, HSQC, and HMBC. As shown in Figure 5, the ^1^H and ^13^C NMR spectra of these three GUPSs were crowded in a narrow region within 3.0–5.3 ppm (^1^H NMR) and 60–110 ppm (^13^C NMR), which are typical characteristics of polysaccharides [43,44]. Anomeric proton signals at δ 5.09, 5.23, and 5.39 ppm, and anomeric carbon signals at δ 99.69, 95.2, and 92.24 ppm were observed in GUPS-I (Figure 5A,B), which revealed that it might be composed of three types of monosaccharide residues, and only contained α-glycosidic linkage. This is consistent with its monosaccharide composition. According to the ^1^H-^13^C HSQC (Figure 6A) and HMBC (Figure 6B) spectra, the signals were very weak, indicating that the structure of GUPS-I was relatively simple. However, the ^1^H NMR and ^13^C NMR spectra of the GUPS-II and GUPS-III revealed that they contained both β-glycosidical and α-glycosidical configurations (Figure 5C–F). The ^1^H NMR spectrum of GUPS-II contains nine main anomeric proton signals at δ 5.28, 5.26, 5.13, 5.06, 5.03, 4.97, 4.85 4.53, and 4.52 ppm, while GUPS-III contained six main anomeric proton signals at δ 5.66, 5.58, 5.13, 5.03, 4.97, and 4.36 ppm. The proton signals at δ 5.28 in GUPS-II and δ 5.58 ppm in GUPS-III indicated the presence of α-l-Ara. The proton signal at δ 5.26 ppm indicated the presence of α-l-Rhap in GUPS-II but not in GUPS-III. This was consistent with their monosaccharide composition (Figure 3). But the signals at δ 1.12–1.19 ppm in GUPS-III indicated the presence of methyl group of Rha. Weak signals at around δ 2.0 ppm (2.02 and 1.99 ppm) in GUPS-III were associated with acetyl groups binding at O-2 and O-3 of GalpA, respectively. Signals at approximately δ 175 ppm in ^13^C NMR spectrum from GUPS-III further proved that the α-d-GalpA was partly methyl esterified. This is in accordance with the FT-IR spectrum of GUPS-III, which contained a strong absorption at 1615 cm^−1^. According to HSQC spectra (Figure 6C,E), the anomeric proton and carbon signals were analyzed as follows. Signals at δ 4.98/107.43, 4.92/107.32, 5.21/99.82, 5.29/99.63, and 4.51/95.62 in GUPS-II were assigned to the H-1/C-1 of α-l-Ara*f*-(1-, 3)-α-l-Rha-(1-, 3)-α-d-Gal*p*-(1-, α-d-Xyl*p*-(1-, and -4)-α-d-Glc*p*-(1- residues, respectively. Signals at δ4.98/107.37, 4.96/107.31 in GUPS-III were assigned to the H-1/C-1 of α-l-Ara*f*-(1-, 3)-α-l-Rha-(1- residues, respectively. The HMBC was used to determine the sequence of these residues in the repeating unit and to verify the assignments from HSQC [45]. The HMBC spectrum of GUPS-II was different to that of GUPS-III, suggesting that GUPS-II and GUPS-III have different structural characteristics (Figure 6D,F).

### 3.6. GUPS Promotes the Maturation of DCs

DCs are professional antigen-presenting cells with the ability to initiate immune responses. Recently, lots of effort has been focused on the immunomodulatory activities of natural polysaccharides, especially in DC maturation and function [10]. With the maturation of DCs, the phagocytosis activity was decreased. To evaluate the effect of GUPS on the phagocytosis of DCs, the cells were incubated with FITC-dextran after GUPS treatment and analyzed by flow cytometer. Compared with untreated DCs, the frequencies of Dextran^+^ DCs were significantly reduced by the treatment of LPS, GUPS-I, GUPS-II, and GUPS-III (Supplementary Figure S2), indicating that the three fractions of GUPS promoted DC maturation.

**Figure 7:**
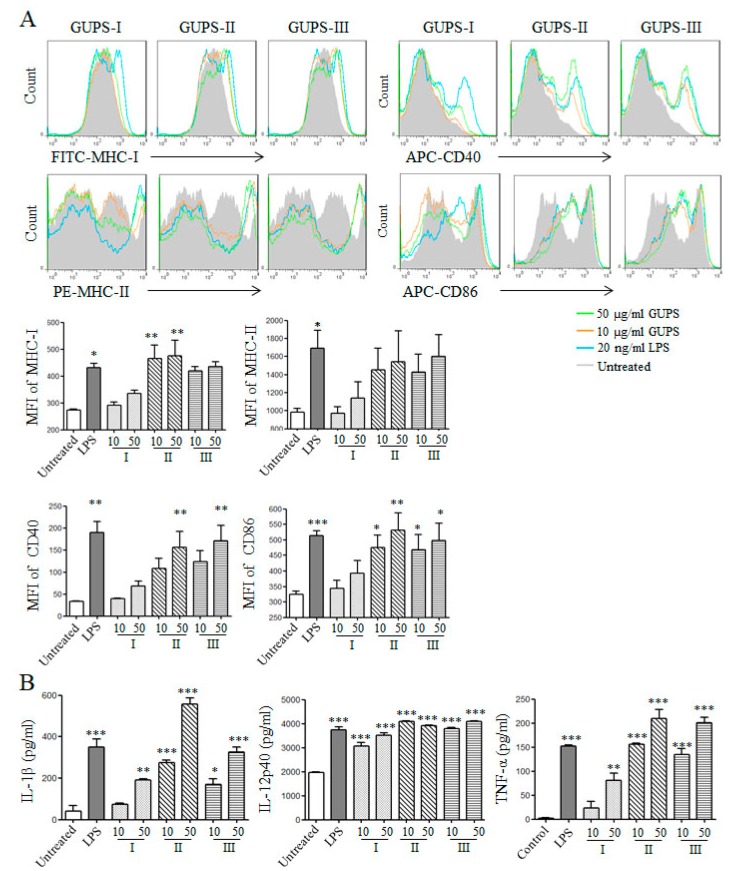
The effects of GUPS fractions on the maturation and cytokine production of DC in vitro. On day 7, bone marrow-derived DCs were treated with different concentrations (10 and 50 μg/mL) of GUPS for 12 h. (**A**) After treatment, the expressions of CD40, CD86, MHC I, and MHC II on DCs were detected by flow cytometry (upper panels). The mean fluorescence intensity (MFI) (mean ± SEM) is shown in lower panels. (**B**) The supernatants were collected and the production of IL-1β, IL-12p40, and TNF-α was detected by ELISA. The concentrations (mean ± SEM) of cytokines are shown. Data are from three independent experiments and analyzed by ANOVA. * *p* < 0.05; ** *p* < 0.01; *** *p* < 0.001 compared with untreated DCs.

**Figure 8:**
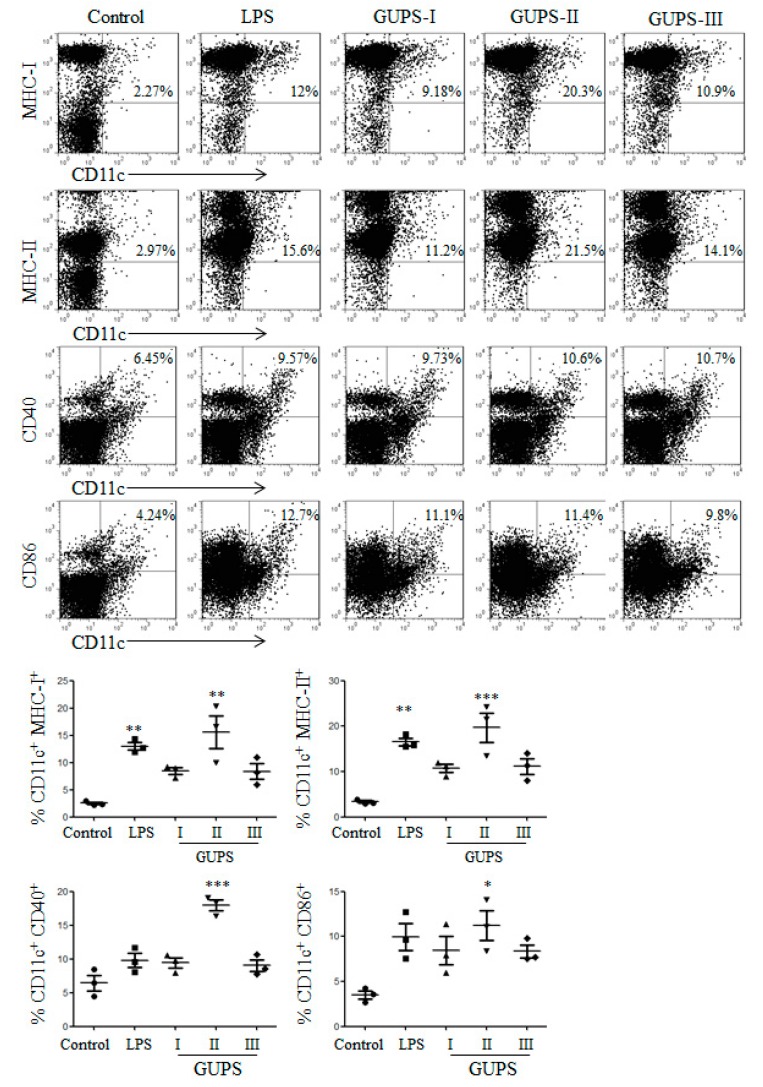
The effects of GUPS fractions on DC maturation in vivo. Mice were injected with GUPS-I, GUPS-II, or GUPS-III by footpads. After 24 h, popliteal LNs were isolated to analyze DC maturation by flow cytometry (upper panels). The MFI (mean ± SEM) is shown in lower panels. Data are analyzed by ANOVA. * *p* < 0.05; ** *p* < 0.01; *** *p* < 0.001 compared with the controls.

We further investigated the phenotype maturation and cytokine production of DCs both in vitro and in vivo after the treatment of GUPS-I, GUPS-II, and GUPS-III. GUPS-I, GUPS-II, and GUPS-III significantly enhanced the expression of MHC-I, CD40, and CD86 (Figure 7A). All three fractions significantly increased the secretion of IL-1β, IL-12p40, and TNF-α. GUPS-II and GUPS-III showed stronger activity than GUPS-I in the cytokine production (Figure 7B). Moreover, the in vivo experiment showed that only GUPS-II significantly increased the frequencies of CD11c^+^CD40^+^, CD11c^+^CD86^+^, CD11c^+^MHC-I^+^, and CD11c^+^MHC-II^+^ cells in LNs (Figure 8), suggesting that only GUPS-II could promote DC maturation in vivo. The results suggested that GUPS-II has stronger immuno-enhancing activity than GUPS-I and GUPS-III, which might be due to the high molecule weight and polysaccharide content of GUPS-II.

### 3.7. Diphenylpicrylhydrazyl (DPPH) Radical Scavenging Activity of GUPS

The antioxidative activities of GUPS-I, GUPS-II, and GUPS-III were detected by DPPH radical scavenging assay. All three GUPS fractions showed the DPPH radical scavenging activities. GUPS-II and GUPS-III showed stronger antioxidative activities than that of GUPS-I (see Supplementary Figure S3). 

## 4. Discussion

Three water-soluble polysaccharides, GUPS-I, GUPS-II, and GUPS-III, with molecular weights of 1.06 kDa, 29.1 kDa, and 14.9 kDa, respectively, were isolated from the herbal medicine *G. uralensis* via DEAE-52 ion-exchange chromatography. GUPS-I is a white granule and only contains α-glycosidic bonds. GUPS-II and GUPS-III are flaky with a smooth surface and contain α- and β-glycosidic linkages. GUPS-III appears to be sulfated polysaccharides. GUPS-I might be a neutral polysaccharide because it coeluted with distilled water, whereas both GUPS-II and GUPS-III, which eluted with NaCl solution, might be acidic polysaccharides [29]. The results are similar to previous studies which reported that three major fractions can be obtained from crude polysaccharides of *G. uralensis* [30,31], but different from another study which reported that five fractions can be observed [46]. 

These three fractions were analyzed qualitatively using HPGPC, GC-MS, SEM, FT-IR, and ^1^H-NMR. The monosaccharide composition of GUPS-II is similar to that reported in previous studies, while GUPS-III has some differences, such as its mannose content [30,31]. GUPS-I and GUPS-II do not contain any protein, whereas GUPS-C and GUPS-III contain 4% and 21.5% protein, respectively. We speculate that GUPS-C may contain some glycoproteins which may be enriched in the GUPS-III fraction. The sulfate contents of GUPS-I (6.5%) and GUPS-II (8.7%) are similar to those reported in previous studies, while the sulfate content of GUPS-III (20.47%) is much higher than that in previous reports (5.43% and 3.42%) [29,30]. GUPS-II and GUPS-III are flaky without a porous structure, which is in contrast to data from a previous study [31]. GUPS-II and GUPS-III both show a smooth surface, suggesting that they may form an amorphous structure. This is also in contrast to what was reported the previous study mentioned above [31]. These results indicated that the physicochemical properties of these three GUPS fractions (extracted from *G. uralensis* from Xinjiang, China) are different from those polysaccharides purified by other groups from the same species but grown in different locations in previously reported studies (*G. uralensis* from Ningxia and from Gansu, China) [30,31]. The biotopes of *G. uralensis* from different places may cause the difference of polysaccharide accumulation, as well as the function of polysaccharides.

It has been reported that polysaccharides of *G. uralensis* have antioxidant activities [30,31,46]. Similarly, we also found that all three GUPS fractions have antioxidant activities. However, the antioxidant activity of GUPS-I is lower than that of GUPS-II and GUPS-III, which is different from what was reported in a previous study [30]. This may be due to the low molecular weight and carbohydrate content of GUPS-I. 

Our previous study showed that GUPS-C enhances the maturation and function of DCs and the antitumor efficacy of DC-based vaccine [23]. The immuno-enhancing activities of three GUPS fractions with undetectable levels of endotoxin were further investigated using the in vitro-cultured DCs and a mouse model in the current study. We showed that GUPS-II and GUPS-III exhibit stronger activity than that of GUPS-I in the induction of DC maturation and cytokine production in vitro. Compared with GUPS-I and GUPS-III, GUPS-II showed significantly enhanced DC maturation in vivo, suggesting that it has the potential to be used along with vaccine or cancer immunotherapy as an immune-stimulator.

In conclusion, three water-soluble polysaccharides were isolated from the herbal medicine *G. uralensis* grown in Xinjiang. These three fractions have different physicochemical properties and different antioxidant and immuno-enhancing activities. GUPS-II has the strongest immuno-enhancing activity, which makes it a potential candidate to be developed into an immune-enhancing adjuvant for cancer immunotherapy.

## Figures and Tables

**Table 1:** Physicochemical properties of GUPS.

Samples	GUPS-C	GUPS-I	GUPS-II	GUPS-III
Color	Brown	White	Faint yellow	Yellow
Texture	Granulate	Granulate	Loose	Loose
Water solubility	Better	Good	Good	Good
Molecular Weight (Da)	nm	1060	29100	14900
Carbohydrate content (%)	37.7 ± 0.34	29.1 ± 0.14	93.5 ± 0.68	75.2 ± 0.54
Protein content (%)	4.1 ± 0.04	0	0	21.5 ± 0.02
Sulfate content (%)	nm	6.5 ± 0.45	8.7 ± 0.23	20.5 ± 0.76

nm: Not measured.

## Supplementary Materials

The following are available online at https://www.mdpi.com/2218-273X/10/1/159/s1, Figure S1: The UV spectra of GUPS-I, GUPS-II and GUPS-III, Figure S2: The capacity of antigen up-take of DCs upon GUPS treatment, Figure S3: The antioxidant activities of GUPS fractions.
