# Supplementary material for: Preparation, Characterization, and Immuno-Enhancing Activity of Polysaccharides from Glycyrrhiza uralensis
Source: Biomolecules. 2020 Jan 19;10(1):159. doi: 10.3390/biom10010159 (PMC7022281; doi:10.3390/biom10010159)
Supplement: Supplementary file 1 [file biomolecules-10-00159-s001.pdf]

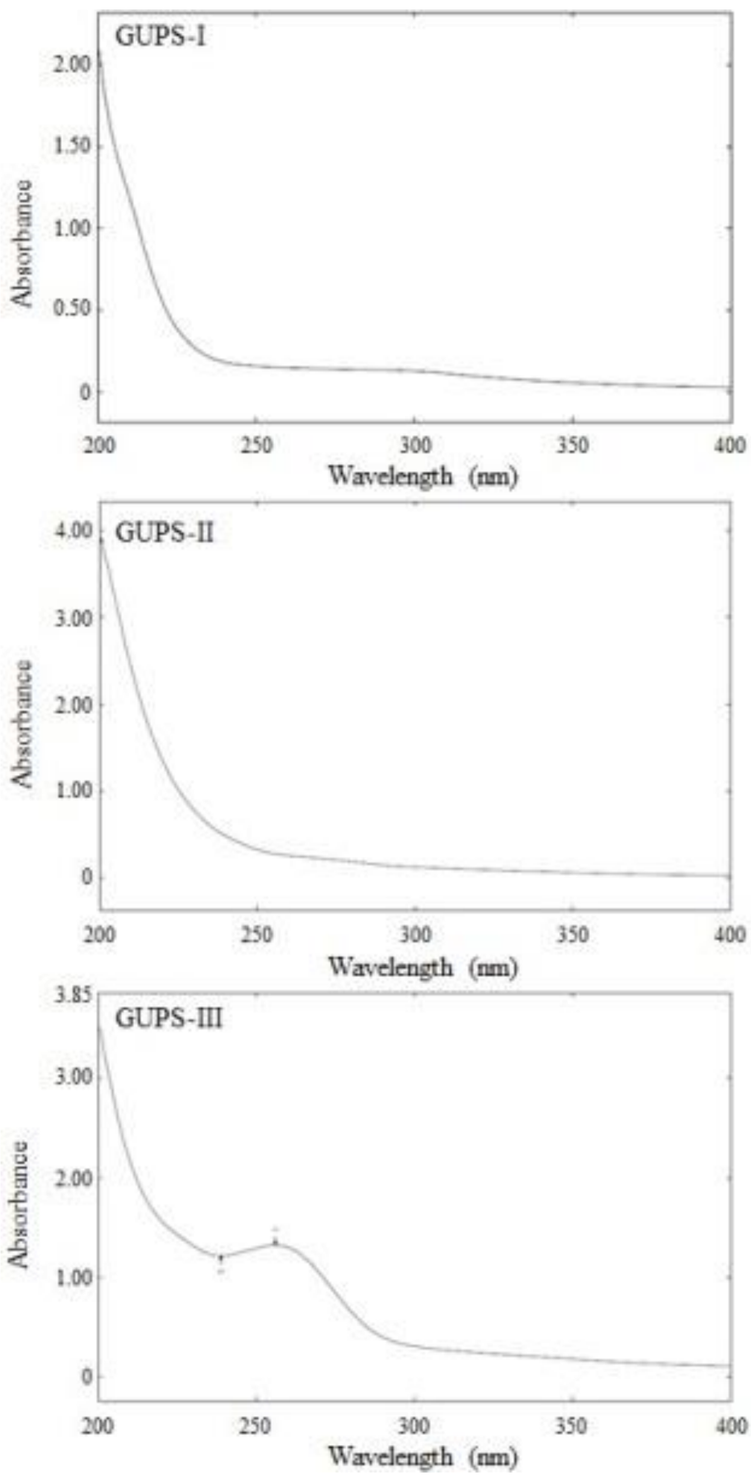

Supplementary Figure S1. The UV spectra of GUPS-I, GUPS-II and GUPS-III.

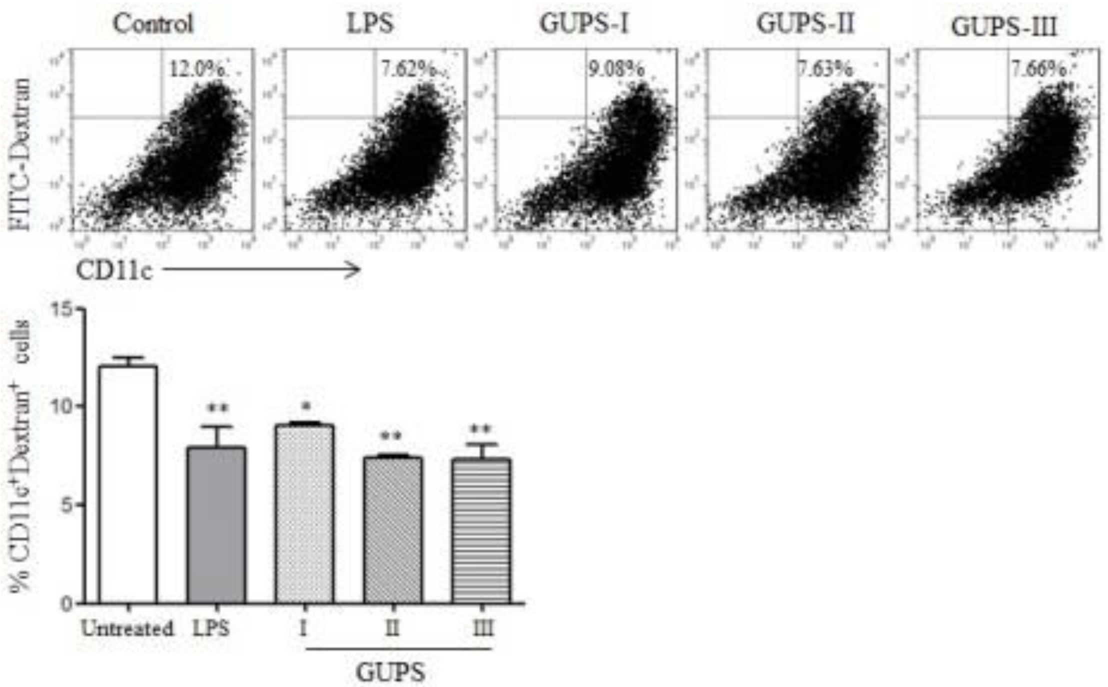

Supplementary Figure S2. The capacity of antigen up-take of DCs upon GUPS treatment. DCs were treated with GUPS fractions (50 μg/mL) for 12 h, and then inoculated with FITC-Dextran for 1 h. After staining with PE-CD11c, samples were analyzed by flow cytometry (upper panels). The summary data (mean ± SEM) are shown in lower panel. Data are from 3 independent experiments and analyzed by ANOVA. * $p < 0.05$; ** $p < 0.01$ compared to untreated DCs.

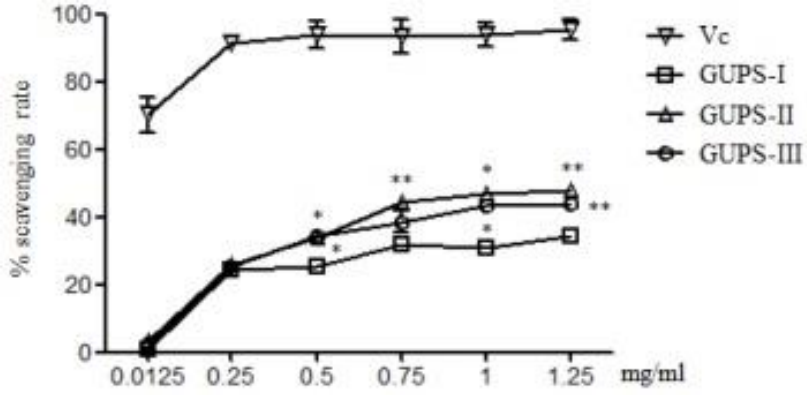

Supplementary Figure S3. The antioxidant activities of GUPS fractions. Vc was used as positive control. Data are from 3 independent experiments, the two-tailed unpaired t-test was used to compare GUPS-II and GUPS-III with GUPS-I. * $p < 0.05$; ** $p < 0.01$.
